# Better and more efficient care through ICT-enabled integration of social care and healthcare services: experiences from two European projects

**Published:** 2012-06-15

**Authors:** Sonja Müller, Ingo Meyer, Lutz Kubitschke, Sarah Delaney

**Affiliations:** Empirica GmbH, Germany; Empirica GmbH, Germany; Empirica GmbH, Germany; Work Research Centre, Ireland

**Keywords:** integrated care, ICT, telecare, telehealth, service innovation

## Abstract

Unsynchronised social and health care service delivery leads to inefficiencies, duplication of resources and reduced levels of quality of care. Older people are particularly affected by this situation. They often need both types of services, such as support with daily living activities and chronic disease management. ICT has the potential to support integrated service delivery to achieve high quality independent living and wellbeing for older people across Europe and elsewhere. Against this background, the presentation will demonstrate experiences and results derived from the development and piloting of ICT-supported integrated care services in eight sites across Europe, namely Dublin, Hull, Milton Keynes, Malaga, Veldhoven, Geldrop, Eindhoven and Bielefeld. Through innovative usage of ICT, current ‘silos’ in service delivery are broken up to allow for cooperation across relevant care sectors and participation of family members. The integrated services are to support the effective management of chronic diseases, and to address issues which affect independence, such as reduced agility, vision or hearing, in order to significantly improve the quality of life for older people and their carers.

A dedicated programme of service process innovation complemented by adaptation of technology is being pursued in order to develop an integrated digital support infrastructure and related services:

- using appropriate existing technology to provide as many older people as possible with digital access to support services they need
- augmenting and opening sectoral care platforms to enable coordinated cross-sector support delivery
- adopting a clearly demand-driven inclusive approach and avoiding a technology ‘push’.

Wider deployment of the services is supported by a dedicated programme of socio-economic service evaluation. The evaluation framework utilises a multi-method and multi-perspective approach, involving end users, family carers, service provider staff and key informants at corporate level. Triangulation is used to cross-reference data from different sources in order to maximize the reliability and robustness of conclusions drawn from the evaluation. Based on an overall framework taking into account themes such as integration, user outcomes, staff impact, organisational impact, technology, implementation and overall satisfaction, the specifics of each site are taken into account in operationally applying the overall framework in each case. The designs to be employed at each site have been developed to be as robust as possible, taking into account the constraints of the realities of the interventions.

The evaluation is accompanied by a business case modelling approach that builds largely on a cost-benefit analysis covering the service development and implementation activities as well as the pilots and modelling the further deployment of services in each of the pilot sites. The presentation builds upon experiences gained within the framework of two European projects, CommonWell and INDEPENDENT. They are both co-funded under the EU’s Competitiveness and Innovation Framework Programme (CIP) focus on better joining-up of formal social/healthcare services and strengthening participation of the so-called ‘third sector’.

